# Exoskeleton technology in nursing practice: assessing effectiveness, usability, and impact on nurses’ quality of work life, a narrative review

**DOI:** 10.1186/s12912-024-01821-3

**Published:** 2024-03-05

**Authors:** Alexandre Vallée

**Affiliations:** Department of Epidemiology and Public Health, Foch Hospital, 92150 Suresnes, France

**Keywords:** Nurse, Exoskeleton, Quality of work life, Robots, Fatigue, Pain, Work performance, Nursing practice

## Abstract

The use of exoskeletons in nursing practice has gained attention as a potential solution to address the physical demands and risks associated with the profession. This narrative review examines the effectiveness, usability, and impact of exoskeleton technology on nurses’ quality of work life. The review focuses on the reduction of physical strain and fatigue, improved posture and body mechanics, enhanced patient care, usability and acceptance factors, and the broader impact on work life. The effectiveness of exoskeletons in reducing physical strain and fatigue among nurses is supported by evidence showing decreased muscle activation and reduced forces exerted on the body. The usability and acceptance of exoskeletons are critical considerations, including device comfort and fit, ease of use and integration into workflows, user experience and training, compatibility with the work environment, and user feedback for iterative design improvements. The implementation of exoskeletons has the potential to positively impact nurses’ work life by reducing work-related injuries, improving physical well-being, enhancing job satisfaction, and promoting psychological and psychosocial benefits. Additionally, the use of exoskeletons can lead to improved patient care outcomes. Challenges and future directions in the field of exoskeleton technology for nurses include cost and accessibility, adaptability to nursing specialties and tasks, long-term durability and maintenance, integration with personal protective equipment, and ethical considerations. Addressing these challenges and considering future research and development efforts are crucial for the successful integration of exoskeleton technology in nursing practice, ultimately improving nurses’ quality of work life and patient care delivery.

## Introduction

Nursing is a physically demanding profession that often requires nurses to perform repetitive and strenuous tasks, leading to increased risk of work-related injuries and physical fatigue [1, 2]. To address these challenges and improve nurses’ quality of work life, innovative technologies such as exoskeletons have emerged as potential solutions [3]. Exoskeletons are wearable devices designed to provide mechanical support and assistance to the user, augmenting their physical capabilities and reducing the strain on the body [4]. The assessment of exoskeletons on nurses’ quality of work life is a topic of growing interest and research [5, 6]. Understanding the impact of exoskeleton technology on nurses’ well-being, job satisfaction, and overall work performance is crucial for their successful integration into nursing practice [7].

The effectiveness of exoskeletons in reducing physical strain and fatigue among nurses is a key aspect to be examined [5]. Assessing the impact of exoskeletons on nurses’ biomechanics, such as posture and muscle activity, can provide insights into the potential benefits of these devices in alleviating the physical demands of nursing tasks [8]. Additionally, understanding the usability and acceptance of exoskeletons among nurses is essential to ensure their practicality and integration into daily practice [9].

Furthermore, it is crucial to explore the broader impact of exoskeletons on nurses’ work life. This includes examining their effects on job satisfaction, physical well-being, and overall work performance [5]. Understanding how exoskeletons influence nurses’ productivity, engagement, and overall job satisfaction can provide valuable insights into the potential benefits of these devices in enhancing nurses’ work life [10]. This narrative review aims to provide a comprehensive analysis to shed light on the potential benefits and challenges associated with the use of exoskeletons in improving nurses’ quality of work life.

## Search strategy

PubMed Medline Web of Science, Google Scholar, Scopus databases were used for the research, with only articles in English language, using the following terms: “Exoskeleton”, “Nurse” and “Nursing”. Articles included in this review were both, original research, reviews, viewpoints, and opinions articles. Literature was searched from inception to 2023.

### Definition of exoskeletons

Wearable exoskeletons (*exos*) enhance traditional ergonomic solutions, potentially decreasing both lower back disorders and various work-induced musculoskeletal issues stemming from overexertion. The term ‘*exos*’ encompasses a diverse range of devices, extending from unpowered and semi-passive mode-switching devices to fully powered systems, and includes both stiff exoskeletons and more pliable exosuits. Studies have demonstrated that *exos* can diminish musculoskeletal strain [11–13], significantly mitigating a primary contributor to overexertion injuries in occupational settings [14]. While some pioneers in the field have labeled *exos* as personal protective equipment, others view them as engineering tools or controls [15]. They fall into various categories, primarily distinguished by their operational mechanisms and structural design: active, passive exoskeletons and, hard and soft robotics [16]. Active exoskeletons devices are motorized and often incorporate advanced technologies like sensors, actuators, and control algorithms. These devices are powered, typically by batteries, and can provide significant physical assistance or augmentation. They are particularly useful in heavy lifting, rehabilitation, or enhancing the strength and endurance of the user. The active components actively work to support, enhance, or supplement the user’s movements. Unlike their active counterparts, passive exoskeletons do not have motorized parts. They rely on mechanical means such as springs, dampers, and elastic elements to assist the wearer. Passive exoskeletons are typically lighter, less complex, and do not require an external power source. They are often used to reduce strain in specific body parts, improve posture, or alleviate fatigue in repetitive tasks. Hard robotics refer to exoskeletons made from rigid materials like metal or hard plastics. Hard robotic exoskeletons are more common in scenarios requiring heavy-duty support or protection, such as in industrial applications or in military usage. They provide a high level of structural support and are often more durable. Soft robotic exoskeletons, or exosuits, are made from flexible, often textile-based materials. They are lighter and offer more flexibility and comfort compared to hard exoskeletons. Soft robotics is an emerging field and these exoskeletons are gaining popularity in medical rehabilitation and assistance for everyday tasks, as they offer a more natural range of motion while still providing support and augmentation. Each type of exoskeleton present different advantages and disadvantages (Table 1).

**Table 1:** Advantages and disadvantages of exoskeletons

	Advantages	Disadvantages
Active exoskeletons	• Significant physical support, augmenting the user’s strength and endurance. • Incorporate sensors, actuators, and control algorithms, allowing for sophisticated and responsive assistance. • Adjust the level of assistance based on the task or the user’s movement, offering personalized support.	• High Energy Consumption • High cost • Eight and bulkiness • Maintenance complexity • Potential for Over-Reliance
Passive exoskeletons	• Suitable for all-day use • Simple to clean • User-friendly • Affordable	• Offer less support compared to powered exoskeletons.
Hard exoskeletons	• Offers ample support during weightlifting activities. • Automatically adapts to provide the necessary level of assistance.	• Driven by high energy demands (limited endurance, significant energy usage). • Larger, more cumbersome design (reduced adaptability to various environments). • Rigid framework (hinders daily activities, restricts movement) • Maintenance can be challenging. • High cost
Soft exoskeletons	• Conforms well to the body’s shape. • Easily customizable for a comfortable fit. • Compact and lightweight design. • Compatible for wear under personal protective equipment (PPE). • Cost-effective	• Challenging to attach motors and sensors. • Absence of a rigid structure leads to reduced strength support. • Places strain on the body

### Effectiveness of exoskeletons

The effectiveness of exoskeletons in reducing physical strain and fatigue among nurses has been a subject of investigation in several studies [5, 8, 17, 18]. These wearable robotic devices are designed to provide mechanical support and assistance, aiming to alleviate the physical demands of nursing tasks and mitigate the risk of musculoskeletal injuries. The following sections explore the evidence regarding the effectiveness of exoskeletons in improving nurses’ work life.

#### Reduced physical strain

Exoskeletons have shown promising results in reducing physical strain on nurses during patient handling tasks, such as lifting and transferring patients [5, 8]. These devices are designed to provide support to the lower back, upper body, and extremities, reducing the load on the musculoskeletal system. Studies have reported decreased muscle activation and reduced forces exerted on the body when nurses wear exoskeletons, suggesting a potential reduction in the risk of musculoskeletal injuries [15].

#### Improved posture and body mechanics

One of the primary benefits of exoskeletons is their ability to promote improved posture and body mechanics [19, 20]. These devices are designed to encourage proper alignment and movement patterns, which can help prevent excessive stress on the joints and muscles. By providing external support and guidance, exoskeletons can help nurses maintain ergonomic positions during physically demanding tasks, reducing the strain on their bodies and potentially minimizing the risk of injuries. While exoskeletons are designed to support ergonomic postures, they can also restrict natural movement. This limitation can lead to discomfort or even contribute to the development of other musculoskeletal issues [21]. Long-term reliance on exoskeletons for posture support might lead to a reduction in the natural strength and conditioning of the body’s musculoskeletal system, potentially making nurses more prone to injury when not using the device.

#### Reduced physical fatigue

Nursing work often involves repetitive and physically demanding tasks that can lead to fatigue over time [22–24]. Exoskeletons have shown promise in reducing physical fatigue among nurses by offloading some of the workload from the body’s musculoskeletal system [11]. By providing mechanical support and assistance, these devices can help nurses conserve energy and reduce the overall physical exertion required during patient handling activities [25]. This reduction in physical fatigue can contribute to improved endurance and performance, ultimately enhancing nurses’ quality of work life. Nevertheless, this topic remains few investigated and future research are needed to better show the physical fatigue among nurses by using exoskeletons.

#### Enhanced patient care

The potential benefits of exoskeletons extend beyond the well-being of nurses themselves [3, 26]. By reducing physical strain and fatigue, exoskeletons may enhance nurses’ ability to provide safe and high-quality patient care [8]. When nurses are less physically fatigued, they may be able to maintain better focus, concentration, and precision during patient interactions and procedures [27]. This can potentially lead to improved patient outcomes and overall satisfaction with the care received.

It is important to note that while studies have shown positive outcomes regarding the effectiveness of exoskeletons, the evidence base is still evolving [28]. The available research primarily consists of small-scale studies and simulations, and there is a need for larger-scale, well-designed randomized controlled trials to provide more robust evidence [5]. Additionally, the effectiveness of exoskeletons may vary depending on factors such as the specific design of the device, the type of nursing tasks performed, and individual user characteristics [5, 29].

Exoskeletons have shown promise in reducing physical strain, improving posture, and reducing fatigue among nurses [5, 26]. These devices have the potential to enhance nurses’ work life by minimizing the risk of musculoskeletal injuries and promoting ergonomic practices. However, further research is needed to establish the long-term effectiveness of exoskeletons and their impact on improving patient outcomes and overall healthcare delivery.

### Usability and acceptance

The usability and acceptance of exoskeletons among nurses are critical factors that influence their integration into clinical practice [30]. The successful adoption of exoskeleton technology relies on factors such as device comfort, ease of use, compatibility with nursing tasks and workflows, and user experience [9]. The following sections explore the aspects of usability and acceptance of exoskeletons in the context of nursing.

#### Device comfort and fit

Exoskeletons need to be comfortable for nurses to wear for extended periods [31, 32]. Factors such as device weight, adjustability, and ergonomic design play a crucial role in ensuring comfort. Lightweight and properly fitted exoskeletons can help prevent discomfort, skin irritation, and restricted mobility, allowing nurses to move freely and perform their tasks without hindrance. However, current exoskeleton designs may not support all the tasks performed by nurses. They are often optimized for specific movements or tasks, which means that they might not be beneficial for all aspects of nursing work [8, 33].

#### Ease of use and integration

Exoskeletons should be designed with intuitive controls and easy donning and doffing mechanisms [34]. Nurses need to be able to quickly and efficiently put on and remove the exoskeletons, considering the fast-paced nature of their work. Seamless integration with nursing tasks and workflows is also essential. Exoskeletons should not impede or interfere with the performance of nursing duties, allowing nurses to move naturally and comfortably while wearing the device [35].

While exoskeletons are designed to reduce physical strain, they can also be cumbersome and uncomfortable for some users [11]. The added weight and rigidity of the device might restrict movement, which is critical in a dynamic environment like healthcare [5]. This can lead to additional physical strain or even new types of musculoskeletal injuries.

#### User experience and training

Positive user experience is crucial for the acceptance and adoption of exoskeletons among nurses [5, 13, 32]. Nurses should feel confident and supported while using the device, with clear instructions and feedback on its operation. Comprehensive training programs should be provided to nurses to ensure they understand how to use the exoskeleton effectively and safely. Ongoing support and opportunities for feedback and troubleshooting can contribute to a positive user experience, leading to greater acceptance and integration into daily practice. While exoskeletons aim to assist with physical tasks, their design may not always align perfectly with the ergonomic needs of all users. Discomfort, restricted mobility, or a poor fit can lead to reduced acceptance and reluctance to use the technology regularly. Exoskeletons may inadvertently interfere with certain nursing tasks, especially those requiring fine motor skills or close physical interaction with patients. This can hinder the performance of nurses and affect the overall workflow [8].

#### Compatibility with work environment

Exoskeletons should be compatible with the specific demands and requirements of different nursing specialties and work settings [5, 26, 36]. For example, devices designed for general patient handling tasks may not be suitable for specialized units such as intensive care or operating rooms. Customizable and adaptable exoskeletons that can be tailored to the unique needs of various nursing roles and environments can enhance usability and acceptance. Nevertheless, the complexity of operating exoskeletons can be a significant barrier. Nurses may require extensive training to use them effectively, and the learning curve associated with these devices can be steep, potentially leading to frustration and reduced acceptance.

#### User feedback and iterative design

Incorporating user feedback into the design and improvement of exoskeletons is crucial for enhancing usability and acceptance [37, 38]. Continuous collaboration and communication with nurses who use the devices can provide valuable insights and help address any issues or concerns. User-centered design approaches, such as involving nurses in the development and evaluation of exoskeleton technology, can lead to devices that better meet their needs and preferences [39].

While exoskeletons offer potential benefits, concerns and challenges related to usability and acceptance need to be addressed. Nurses’ acceptance of exoskeletons can be influenced by factors such as perceived stigma, concerns about the impact on professional identity, and the need for clear communication and education to dispel misconceptions [40, 41]. Collaborative efforts involving nurses, researchers, and manufacturers are essential to address these challenges and ensure that exoskeletons are designed and implemented in a way that optimizes usability and acceptance.

The usability and acceptance of exoskeletons among nurses are crucial for their successful integration into nursing practice [42]. Comfort, ease of use, compatibility with tasks and workflows, positive user experience, and ongoing support and training are key considerations. By addressing these factors, exoskeleton technology can be optimized to enhance nurses’ work life and improve the delivery of patient care.

The psychological impact of wearing robotic devices, such as feeling of dependency or altered self-perception, can affect the acceptance of exoskeletons among nursing staff [43]. Resistance to change and the preference for traditional methods of work can also hinder the effective integration of this technology.

### Impact on work life

The implementation of exoskeletons in nursing practice has the potential to significantly impact nurses’ quality of work life [44]. Beyond the physical benefits of reducing strain and fatigue, exoskeletons can have broader implications for job satisfaction, well-being, and overall work performance. The following sections explore the potential impact of exoskeletons on nurses’ work life.

#### Reduced work-related injuries

Exoskeletons have the potential to minimize the risk of work-related musculoskeletal injuries among nurses [33]. By providing mechanical support and assistance during physically demanding tasks, these devices can help alleviate the strain on nurses’ bodies. The reduction in work-related injuries can lead to decreased absenteeism, lower healthcare costs, and a safer work environment for nurses [45]. While exoskeletons can assist with lifting and moving, they do not necessarily address other common causes of work-related injuries in nursing, such as slips, trips, and falls, or injuries sustained during patient assaults. Moreover, there is a learning curve associated with using exoskeletons properly [28]. Improper use or overdependence on the device’s capabilities can lead to misuse, potentially resulting in injuries rather than preventing them. Furthermore, exoskeletons may not be suitable for all body types or for every nurse’s physical condition. This lack of customization can lead to discomfort or even injury if the exoskeleton does not fit properly [5].

#### Improved physical well-being

Enhanced physical well-being is a direct outcome of using exoskeletons [46]. By reducing the physical strain and fatigue associated with nursing tasks, these devices can contribute to improved endurance, reduced muscle soreness, and increased overall physical comfort. Nurses who experience less physical discomfort and fatigue are more likely to feel better equipped to perform their duties effectively and experience less work-related physical stress [47]. Exoskeletons, while designed to reduce strain, can themselves be uncomfortable or restrictive. The additional weight and rigidity of the device might limit natural movement, potentially leading to discomfort or even new types of physical strain [48]. Moreover, there can be an adaptation period when first using exoskeletons. During this time, nurses might experience increased physical discomfort or fatigue as they adjust to the new dynamics of movement and weight distribution [21].

#### Enhanced job satisfaction

Job satisfaction is a crucial component of nurses’ work life, and exoskeletons have the potential to positively impact this aspect [49]. By reducing physical strain and fatigue, nurses may experience a greater sense of control and confidence in their ability to perform their duties. This can lead to increased job satisfaction and a higher overall sense of fulfillment in their professional role. When nurses are satisfied with their work, they are more likely to be motivated, engaged, and committed to providing high-quality care [50, 51]. In contrast, the use of mechanical assistance in patient care might affect the interpersonal dynamics between nurses and patients. The use of a mechanical device in caregiving can lead to feelings of depersonalization, where nurses might feel that their role is being reduced to operating a machine rather than providing holistic care.

#### Psychological and psychosocial benefits

Exoskeletons may also have psychological and psychosocial benefits for nurses [31, 33]. By reducing physical strain and fatigue, these devices can contribute to a reduction in stress levels and an overall improvement in psychological well-being. Nurses who feel physically supported and less fatigued may experience reduced levels of burnout and increased resilience. Additionally, the perception of increased safety and security associated with using exoskeletons can enhance nurses’ confidence and job satisfaction.

#### Impact on patient care

The impact of exoskeletons on nurses’ quality of work life can extend to improved patient care outcomes [52]. When nurses experience less physical strain and fatigue, they are better able to focus on providing safe and effective care to their patients. Enhanced physical well-being can lead to increased attentiveness, accuracy, and precision in performing nursing tasks. Ultimately, this can result in improved patient outcomes, increased patient satisfaction, and a higher quality of care delivery.

However, it is important to acknowledge potential challenges and considerations related to the impact of exoskeletons on work life. Stigma or misconceptions associated with wearing exoskeletons should be addressed through clear communication and education. The physical presence of an exoskeleton could create a barrier or sense of distance between nurses and patients. This might affect the quality of interpersonal interactions, which are crucial in patient care. Malfunctions or technical failures of exoskeletons could disrupt nursing care, potentially leading to delays or errors in patient care. Differences in individual experience with exoskeletons, due to factors like body type and personal preference, could lead to inconsistent impacts on patient care across different nurses. Thus, collaborative efforts among nurses, healthcare organizations, and manufacturers are necessary to ensure that the implementation of exoskeletons supports a positive work environment and fosters acceptance [53].

The use of exoskeletons has the potential to positively impact nurses’ work life by reducing work-related injuries, improving physical well-being, enhancing job satisfaction, and promoting psychological and psychosocial well-being [5]. These devices can also contribute to improved patient care outcomes. Addressing challenges and considerations, along with ongoing evaluation and support, is crucial to maximizing the positive impact of exoskeletons on nurses’ work life and overall healthcare delivery [3].

### Challenges and future directions

While exoskeletons show promise in improving nurses’ quality of work life, several challenges and considerations need to be addressed for their successful implementation in nursing practice. Few investigations have shown interesting results (Table 2); thus, future research and development efforts are essential to further enhance the effectiveness, usability, and acceptance of exoskeletons. The following sections discuss the challenges and future directions in the field of exoskeleton technology for nurses.

**Table 2:** Examples of exoskeleton applications

Year	Exoskeletons	Subjects	Results	Reference
2023	Low Back Exoskeleton	14 nurses	Satisfaction score of the nurses relative to the use of the exoskeleton was 6/10. The median impact of the exoskeleton on nurses’ fatigue was 7/10.	[5]
2023	Qualitative research	8 nurses	Exoskeleton was felt to be easy to use after initial adjustments	[61]
2023	Qualitative research	7 nurses	Five themes emerged from the interviews (workflow, user needs, hindrances, motivation for intervention, and acceptance)	[63]
2022	Passive back-assist exoskeleton	23 nurses	Perceived usefulness and enjoyment of use increases and anxiety toward the use decreases nurses’ exoskeleton acceptance.	[26]
2021	Hybrid assistive limb	19 nurses	Mean lumbar fatigue VAS score for all participants without the HAL for Care Support was 62 mm, while that with it was 43 mm.	[62]
2021	Passive back-support exoskeletons	20 nurses	The muscle activities of the erector spinae were significantly lower (up to 11.2%) compared to no exoskeleton use	[64]
2020	Passive shoulder-assist exoskeleton	4 surgical nurses	Usability scale was 81.3 out of 100	[53]

#### Cost and accessibility

One significant challenge is the cost associated with acquiring and maintaining exoskeletons [28]. Currently, exoskeletons can be expensive, limiting their availability and accessibility in healthcare settings, particularly in resource-constrained environments. Efforts should be made to explore cost-effective options and develop affordable exoskeleton solutions that can be widely adopted [54]. Additionally, partnerships between healthcare organizations and manufacturers can help in addressing financial barriers and ensuring the availability of exoskeletons for nurses.

#### Adaptability to nursing specialties and tasks

Exoskeletons need to be adaptable to the diverse range of nursing specialties and tasks to maximize their usability and effectiveness. Different nursing roles may require specific adjustments and modifications to accommodate the unique physical demands of each specialty. Exoskeletons should be designed to be versatile and customizable, allowing for adjustments to suit various nursing tasks and environments.

#### Long-term durability and maintenance

The long-term durability and maintenance requirements of exoskeletons are important considerations [55]. These devices need to withstand the rigors of daily use in a healthcare setting and be durable enough to withstand frequent cleaning and disinfection. Ensuring that exoskeletons have a long lifespan and minimal maintenance requirements can help reduce the cost and logistical challenges associated with their implementation.

#### Integration with personal protective equipment (PPE)

Exoskeletons should be designed to integrate seamlessly with existing personal protective equipment (PPE) used by nurses, such as gloves and gowns [8, 56]. Compatibility with PPE is crucial to ensure that nurses can continue to perform their tasks safely while wearing exoskeletons. Collaboration between exoskeleton manufacturers and PPE suppliers can help ensure proper integration and optimize the safety and usability of these devices [57].

#### Ethical considerations

Ethical considerations related to the use of exoskeletons in nursing practice should be carefully addressed [58, 59]. Issues such as data privacy, informed consent, and potential unintended consequences need to be considered. Policies and guidelines should be established to ensure that the implementation of exoskeleton technology adheres to ethical standards and protects the rights and well-being of nurses and patients.

Future research should focus on conducting well-designed randomized controlled trials and longitudinal studies to gather robust evidence on the impact of exoskeletons on nurses’ quality of work life, patient outcomes, and healthcare system sustainability. Long-term studies that assess the durability of exoskeletons, their effects on work-related injuries, fatigue, and job satisfaction are crucial for building a stronger evidence base [60]. Additionally, ongoing collaboration between researchers, engineers, healthcare professionals, and manufacturers is needed to refine and improve exoskeleton technology.

## Conclusion

The assessment of exoskeletons on nurses’ quality of work life reveals promising results in terms of reducing physical strain, improving posture, reducing fatigue, and enhancing job satisfaction. Exoskeletons offer potential benefits in terms of reducing work-related injuries, improving physical well-being, and enhancing overall work performance. These devices have the potential to improve nurses’ ability to provide safe and high-quality patient care, leading to improved patient outcomes and satisfaction.

## Data Availability

Not applicable.
